# Water-deficiency conditions differently modulate the methylome of roots and leaves in barley (*Hordeum vulgare* L.)

**DOI:** 10.1093/jxb/erv552

**Published:** 2016-01-05

**Authors:** Karolina Chwialkowska, Urszula Nowakowska, Anna Mroziewicz, Iwona Szarejko, Miroslaw Kwasniewski

**Affiliations:** Department of Genetics, University of Silesia in Katowice, Jagiellonska 28, 40-032 Katowice, Poland

**Keywords:** Barley, DNA methylation, drought, epigenetics, gene expression, water stress.

## Abstract

One of the strategies of plant adaptation to stress is the modulation of gene expression, which may result from the regulation of DNA methylation. This study attempted to characterize and compare the barley methylome of leaves and roots under water-deficiency treatment and in the subsequent rewatering phase. Our results, obtained using methylation-sensitive amplification polymorphism sequencing analysis, indicated that the overall DNA methylation level in the barley genome was high and in general stable under water-deficiency conditions. Nevertheless, numerous differentially methylated sites (DMSs) were induced by stress in the leaves and roots. Equal proportions of novel stress-induced methylation and demethylation events were observed within the genes in the leaves, but new methylations dominated in the roots. Repetitive elements preferentially underwent demethylation in the leaves and novel methylations in the roots. Importantly, rewatering and plant recovery resulted in the reversibility of the majority of stress-induced methylation events, but this process was more efficient in the leaves than in the roots. Different biological processes were enriched within the subsets of the DMSs that were identified in the genic regions of leaves and roots. We assume that the organ specificity of the methylome changes in response to water deficiency might be an important regulatory mechanism that leads to multi-level mechanisms of stress tolerance in barley.

## Highlight

Characterization of barley methylome modulation in leaves and roots under water-deficiency stress and following rewatering with respect to organ-specific responses.

## Introduction

DNA methylation is an epigenetic modification that is the result of the enzymatic addition of a methyl substituent that occurs most frequently on the cytosine base at its fifth position on an aromatic ring. It is widely assumed that cytosine methylation functions mainly as a negative regulator of gene expression and prevents the movement of repetitive elements, thereby maintaining genome integrity. In plants, cytosine can be methylated in three DNA sequences: CG, CHG, and CHH (where H is A, T, or C), with CG methylation being the most frequent (Lister *et al.*, 2008). A dynamic interplay between DNA methylation and demethylation processes is accomplished by specific enzymes. Cytosine methylation in plants is regulated by two complementary mechanisms of maintenance and *de novo* methylation, through an action of DNA methyltransferases families: DNA METHYLTRANSFERASE (MET) and CHROMOMETHYLASE (CMT) acting as maintenance enzymes, and DOMAINS REARRANGED METHYLTRANSFERASE (DRM) specifically involved in *de novo* methylation through an RNA-dependent pathway (Chen *et al.*, 2010). Methylcytosine may be subjected to passive demethylation events during replication, or to the active demethylation resulting from the action of DNA glycosylases that belong to the DEMETER (DME) family (Ooi and Bestor, 2008). In plants, the total 5-methylcytosine content varies among species, ranging from 5.3% (*Arabidopsis thaliana*) to 39.2% (*Narcissus longispathus*), as it is potentially a derivative of genome size and repetitive element content (Alonso *et al.*, 2015). DNA methylation may exhibit tissue specificity regarding its level (Braszewska-Zalewska *et al.*, 2013) and pattern (Zhang *et al.*, 2011; Candaele *et al.*, 2014).

Several methods of methylome analysis are currently used in plant epigenomics, and among them, genome-scale bisulfite sequencing is the most advanced and direct approach (Harrison and Parle-McDermott, 2011). However, this technology is feasible only for model species that have small genomes, as whole-genome bisulfite sequencing is still quite costly, especially for species that have genomes that are dozens of times larger than the genomes of Arabidopsis or rice. Moreover, the presence of DNA methylation at a high level in all of the sequence contexts (CG and CHG, as well as CHH) and the repetitive nature of large cereal genomes make the analysis of data very challenging. For such species, indirect techniques of DNA methylation analysis are widely used due to their simplicity and cost-effectiveness. One of these is methylation-sensitive amplification polymorphism (MSAP), which applies cleavage with methylation-sensitive restriction enzymes *Hpa*II or *Msp*I, followed by adapter ligation, amplification, and further gel-based visualization (Xu *et al.*, 2000). The methylation state of the cytosines within the recognized 5′-CCGG-3′ sequence can be determined for each band based on the capacity of each enzyme to cleave methylated restriction sites. The MSAP method has proven its usefulness in many global analyses of DNA methylation in plants (Filek *et al.*, 2006; Wang *et al.*, 2011; Guzy-Wrobelska *et al.*, 2013; Marconi *et al.*, 2013; Tang *et al.*, 2014). However, it only provides a general overview of methylation events without a sequence context. In order to directly identify the genomic sequences that are undergoing DNA methylation changes, we have modified the MSAP approach by replacing the conventional separation of MSAP amplicons in polyacrylamide gels with their direct sequencing using next-generation sequencing (NGS) methods and automated data analysis. Our MSAP sequencing (MSAP-Seq) technique allows the global and direct identification of a large set of sequences that are undergoing DNA methylation changes without laborious band excisions and subcloning, which is performed occasionally during MSAP analysis, although on a small scale. Changes in DNA methylation that are assessed using MSAP-Seq are characterized not only qualitatively, as in conventional MSAP in which amplicons are scored as either present or absent, but also quantitatively by the fold change values of the abundance of the normalized reads in different samples.

One of the strategies that determines the rapid adaptation of plants to changing environmental conditions is their ability to modulate gene expression, which in turn may result from epigenetic mechanisms such as DNA methylation and histone modifications (the inter-reliant processes). DNA methylation may play a role in the regulation of gene expression, and therefore stress responses, as it was found to be modulated under abiotic stress (Karan *et al.*, 2012; Song *et al.*, 2012; Wang *et al.*, 2014). Several studies have provided new insights into the epigenetic basis of stress adaptation processes in plants; however, they have mainly involved the analysis of model species with small genomes, such as *A. thaliana* and *Oryza sativa* (Boyko *et al.*, 2010; Gayacharan and Joel, 2013; Ferreira *et al.*, 2015). Moreover, the majority of analyses employ the indirect MSAP marker technique, and thus they are rather cursory and do not provide detailed information on the specific genomic sites that are affected by DNA methylation changes. MSAP analysis, which was conducted in rice leaves under nitrogen deficiency, revealed that, although the overall level of DNA methylation was stable, one-fifth of the sites that were detected exhibited changes in cytosine methylation (Kou *et al.*, 2011). Interestingly, experiments conducted in rice and rapeseed under drought and salinity stress, respectively, revealed that demethylation was more abundant in tolerant cultivars, whereas novel methylations were more abundant in sensitive ones (Gayacharan and Joel, 2013; Marconi *et al.*, 2013). Most of the studies were based on the analysis of only one plant organ or whole seedlings, and only a few studies have involved the comparative analysis of different organs under stress conditions. In rice, it was shown that shoots and roots may differ greatly in the level and pattern of DNA methylation (Wang *et al.*, 2011; Karan *et al.*, 2012). Moreover, the response of their methylomes to salinity stress was different, with many more changes occurring in the shoots than in the roots of rice seedlings (Karan *et al.*, 2012).

There is very little information in the literature on studies that deal with the issue of the reversibility of stress-induced DNA methylation changes. For example, in rice that was treated with water-deficiency stress at the heading stage, most of the demethylation events in the leaves and roots were reversible, whereas only half of the new methylation events exhibited reversibility upon recovery (Wang *et al.*, 2011). All of the above presented research results were based on a global MSAP analysis, and there are only a few studies that have involved more direct methods that allow a detailed analysis of plant methylomes under abiotic stress. One of these is a study on DNA methylation changes in whole *A. thaliana* seedlings under polyethylene glycol-simulated drought stress using methylation-sensitive cut counting (Colaneri and Jones, 2013). Another study, which was based on the methylated DNA immunoprecipitation and sequencing, described the analysis of the response of the maize root methylome to lead stress (Ding *et al.*, 2014).

Deciphering the complex mechanisms of stress responses in plants is still, considering climate changes and food security issues, a very important research task in basic plant science and in modern agriculture. The presented study attempted to characterize and compare the modulation of the leaf and root methylome in barley under water-deficiency stress and subsequent rehydration conditions with a special emphasis on organ-specific changes in the DNA methylation pattern. Consequently, we have delivered a comprehensive catalogue of the general properties of the methylome of the barley leaf and root and its alteration under abiotic stress and during the following recovery period. We have also described examples of genes undergoing negatively coupled expressional and DNA methylation regulation under water-deficiency stress. Relatively few works, most of which are presented above, have focused on the impact of abiotic stress on DNA methylation in crops and, to the best of our knowledge, no such detailed studies on the root methylome response have been reported in cereals, except for the one performed in maize roots under heavy-metal stress (Ding *et al.*, 2014). Thus, this study is one of the first such detailed analyses of the methylome response of a crop with a large genome in which a direct and comprehensive analytical method of detection of cytosine methylation changes (using MSAP-Seq) was applied. Moreover, a comparison of DNA methylation alteration in two different organs was performed, not only under stress conditions but also during the following recovery phase. All of these factors contribute to the novelty of the presented research in plant epigenomics studies that involve the stress-response pathways.

## Materials and methods

### Plant material and drought treatment conditions

Water-deficiency stress treatment was applied during the seedling stage under strictly controlled conditions. Barley seeds of the spring barley cultivar ‘Karat’ were sown in Petri dishes that were filled with water-soaked vermiculite and maintained at 4 °C in darkness for 2 d. The seeds were then moved to a greenhouse for germination at 20 °C for 2 d. The germinated seeds that had visible radicles were transferred to pots filled with soil that was a mixture of clay and sand (7:2 ratio) with known physicochemical properties. The soil was previously mixed with the proper amount of water and a nutrient medium, which had been optimized for the used soil type in order to reach 12% volumetric water content (vwc). The soil moisture expressed as vwc was measured every day in each pot using a Time-domain Reflectometer EasyTest (Institute of Agrophysics, Polish Academy of Sciences, Poland) over the entire experiment. Plants were grown in a greenhouse at 20/18 °C for a 16/8h photoperiod and at 200 μE m^−2^ s^−1^ light intensity, which was provided by fluorescent lamps. On day 11 after the pre-germinated seeds were sown in the pots, the soil moisture was decreased in some of the pots by withholding the irrigation in order to reach water-deficit conditions at the level of 1.5–2% vwc (drought-stressed plants). The rest of the pots were maintained under regular irrigation (control plants). When the soil moisture of the water-stressed plants reached 3% vwc, the treated and control pots were moved to a growth chamber and grown under the same controlled conditions except for the temperature regime, which was set at 25/20 °C day/night. Water deficiency was maintained for 10 d and afterwards the plants were moved back to the greenhouse where a normal irrigation (reaching 12% vwc) was applied for 14 d (rewatering phase). Material for DNA and RNA extraction was harvested from the second leaves and roots in three biological replicates at each of the following time points: (i) control conditions, on day 11 after sowing, soil moisture 12% vwc; (ii) upon drought stress, after 10 d of water-deficiency treatment, at 1.5–3% vwc; and (iii) after a rewatering period that lasted 2 weeks under optimal soil moisture conditions (12% vwc). One biological replicate included a sample of one plant grown in a separate pot. The detached tissue samples were frozen immediately in liquid nitrogen and stored at −80 °C until DNA/RNA isolation.

### Nucleic acid isolation

Plant material that was collected at the three time points described above was used for DNA and RNA isolation. Total RNA was isolated using an RNeasy Plant Mini kit (Qiagen) with on-column DNaseI digestion. Genomic DNA was isolated using an OpEx Plant FAST gDNA kit (OpenExome). The yield and purity of the RNA and DNA samples was determined using a NanoDrop ND-1000 spectrophotometer (NanoDrop Technologies). The integrity of the RNA was determined using an Agilent 2100 Bioanalyzer (Agilent Technologies), whereas the integrity of the genomic DNA was evaluated using agarose gel electrophoresis.

### Evaluation of the global DNA methylation pattern using the MSAP method

MSAP marker analysis was performed as described by Filek *et al.* (2006) with slight modifications. Briefly, equal amounts of DNA samples were digested with *Eco*RI/*Msp*I or *Eco*RI/*Hpa*II restriction enzymes (New England BioLabs) for 6h (each in three biological and two technical replicates). The ligation mixture was then added and an adaptor ligation reaction was performed overnight. The ligation products were used for the pre-selective amplification with primers that contained one selective nucleotide (see Supplementary Table S1A available at *JXB* online). After pre-selective amplification, the reaction mixtures were diluted and used for selective amplification with primers containing three selective nucleotides, with *Eco*RI-related primers fluorescently labelled (IRDye 800). Ten different primer combinations were applied. Finally, amplicons were visualized by 6% denaturing PAGE using a Li-Cor automated sequencer (Lincoln). The bands were scored manually in order to determine the presence or absence of the amplification product in both restriction systems and a 0–1 matrix was prepared using Excel software (Microsoft). The methylation state of each site was determined according to Supplementary Table S2 (available at *JXB* online). The site was detected as: type I, unmethylated, when there was a band present for both of the restrictases (*Hpa*II and *Msp*I); type II, hemi-methylated, when there was a band present for *Hpa*II only; type III, symmetrically methylated within the inner cytosine, when there was only a band present for *Msp*I only; and type IV, fully methylated, when there was no band for both of the enzymes (*Hpa*II and *Msp*I).

### Identification of DMSs using the NGS-based MSAP-Seq technique

MSAP-Seq was performed using two sequencing platforms – 454 GS Junior (Roche) and Illumina Hi-Seq 1500 – and therefore slightly different protocols were applied. DNA (250ng) from three independent biological replicates for each organ and time point was cut with the frequently cutting methylation-sensitive restriction enzyme *Hpa*II (New England Biolabs) and the rare-cutting *Eco*RI. Then, appropriate MSAP adaptors were ligated and DNA fragments were amplified with primers that were complementary to the adaptor sequences with one additional selective nucleotide in order to reduce the number of generated fragments (Supplementary Table S1B). Fragments for 454 sequencing were again amplified with primers that contained three additional selective nucleotides, each in five primer combinations. Then, samples from each primer combination and all of the three biological replicates were pooled together. The 454-related fragments were separated using agarose electrophoresis (three lanes per sample) and bands containing fragments ranging from 300 to 550bp were cut out from the gel and extracted using a QIAEX II Gel Extraction kit (QIAGEN). Illumina-related amplicons from three independent biological replicates were pooled together and fragmented using Bioruptor (Diagenode) with pre- and post-fragmentation purification using Agencourt AMPure XP (Beckman Coulter). Sequencing libraries were then prepared using the appropriate kits – a Lib-L preparation (Roche) for 454 sequencing and a NEXTflex Rapid DNA-Seq kit (BIOO Scientific) for Illumina sequencing, each according to the manufacturer’s instructions. The quality of the 454 libraries was determined using a Flashgel system (Lonza) and the quantity was estimated using QuantiFluor^®^-ST (Promega). The quality of the Illumina libraries was analysed using an Agilent Bioanalyzer and an Agilent DNA 1000 kit and their quantities were estimated using a Library Quantification kit – Illumina/LightCycler^®^ 480 (KAPA Biosystems) based on the quantitative PCR (qPCR) reaction. The prepared libraries were sequenced in a 454 GS Junior System (Roche) or Illumina Hi-Seq 1500, accordingly.

Reads were filtered based on the presence of the *Hpa*II-related adapter. Only those reads that had the *Hpa*II-related adapter were preserved for further analysis. These were trimmed and only those with the CGG sequence on the 3′ or 5′ end were kept. Reads of at least 50bp were mapped to *Hordeum vulgare* genome version 082214v1.25 using Bowtie2 (Ensembl Plants release 25). Reads that mapped to the same place in the genome were counted and normalized using reads per million for Illumina reads and reads per 10 000 for 454 reads). Reads were annotated using a gff3 file that had been downloaded from Ensembl Plants (release 25; genome version 082214v1.25) and classified into four groups: genes, promoters, repeats, and intergenic – without annotation. A promoter was defined as a region that was 1000bp that was located upstream of the gene. Differences in the methylation level among the samples were calculated as fold change (FC≥5 for normalized read counts). Gene Ontology (GO) classification and enrichment was performed using Plaza Monocots 3.0 Workbench pipeline (Proost *et al.*, 2015).

A set of sites that were classified as differentially methylated were validated using methylation-sensitive restriction enzyme qPCR (MSRE-qPCR) (Oakes *et al.*, 2006), which is a single-locus DNA methylation analysis. Briefly, equal amounts of genomic DNA were digested overnight with *Hpa*II enzyme. Mock (undigested) samples were treated in the same way but without the addition of a restriction enzyme. All digests were performed in two independent technical replicates for each of the three separate biological replicates from each time point. Diluted samples were further used as templates for qPCR amplification in a Roche LightCycler^®^ 480 System using dedicated manufacturer chemistry and primers flanking restriction cut sites. Relative methylation level was calculated from a difference between crosing-point (*C*_p_) values of the digested and undigested genomic DNA templates.

### Gene expression analysis using RT-qPCR

One microgram of total RNA was used in a reverse transcription (RT) reaction for cDNA synthesis using a Maxima First Strand cDNA Synthesis kit for RT-qPCR (Thermo Scientific). The cDNA that was obtained was diluted 5-fold and used as the template for qPCR amplification in a Roche LightCycler^®^ 480 System using the dedicated manufacturer chemistry. Amplification reactions were performed in two technical replicates and their specificity was determined in a melting-curve analysis. The primers are listed in Supplementary Table S1C. Raw data was processed using LinRegPCR (Ramakers *et al.*, 2003). The reference genes that were used in this study were *ADP-RIBOSYLATION FACTOR 1-LIKE* (*ADP*; MLOC_33833) and *CORNICHON-4* (*COR4*; MLOC_37168). The geometric means of both references were used in further calculations. Relative expression levels were calculated using the Pfaffl formula (Pfaffl, 2001).

### Statistics

Differences in relative methylation or expression levels among samples were analysed using Student’s *t*-test or one-way ANOVA followed by Fisher’s least significant difference (LSD) post-hoc test at *P*≤0.05 using STATISTICA 10 software (StatSoft).

## Results

### General characterization of leaf and root methylome under water deficiency in barley

Global analysis of the barley methylome under water deficiency was performed by applying the MSAP technique. In total 647 and 553 scorable sites (bands on a gel) were analysed in the leaves and roots, respectively. The overall level of DNA methylation within the analysed CCGG sequences (the ratio of the methylated bands to all of the amplified bands) was high, since about 70% of sites that were analysed were in a methylated state in both the barley leaves and roots, regardless of the growth conditions (Table 1). Slight differences regarding the fractions of bands that represented hemi- and fully methylated sites were also observed. Hemi-methylations, representing single CHG or simultaneous CHG and CG asymmetric methylation, were more abundant in leaves than in roots, whereas full methylations, indicating mainly symmetric CG methylation, were more frequent in roots.

**Table 1.** DNA methylation status in barley leaves and roots under specific water conditions

**MSAP band type** ^***^a^***^	**Leaves**			**Roots**		
	**Control**	**Water deficit stress**	**Rewatering**	**Control**	**Water deficit stress**	**Rewatering**
I	180	194	202	162	152	181
II	156	131	152	96	87	79
III	303	273	286	288	298	278
IV	8	49	7	7	16	15
Total analysed bands	647	647	647	553	553	553
Total methylated bands^*b*^	467	453	445	391	401	372
Fully methylated bands^*c*^	311	322	293	295	314	293
MSAP (%)^*d*^	72.1	70.0	68.8	70.7	72.5	67.3
Hemi-methylated ratio (%)^*e*^	24.1	20.3	23.5	17.4	15.7	14.3
Fully methylated ratio (%)^*f*^	48.1	49.8	45.3	53.4	56.8	53.0

^*a*^ Type I is the presence of bands in both *Eco*RI/*Hpa*II and *Eco*RI/M*sp*I digests and indicates the absence of methylation; type II are bands generated in *Eco*RI/*Hpa*II digests but not in *Eco*RI/*Msp*I digests; type III bands appear in *Eco*RI/*Msp*I digests but not in *Eco*RI/*Hpa*II digests; and type IV represents the absence of band in both enzyme combinations

^*b*^ Total methylated bands are II+III+IV bands.

^*c*^ Fully methylated are III+IV bands.

^*d*^ MSAP is a percentage ratio of total methylated bands (II+III+IV) to total amplified bands.

^*e*^ Hemi-methylated ratio is a percentage ratio of total hemi-methylated bands (II) to total amplified bands.

^*f*^ Fully methylated ratio is a percentage ratio of fully methylated bands (III+IV) to total amplified bands.

### Detailed analysis of the barley methylome under water deficiency using large-scale sequencing of MSAP amplicons

Using NGS of MSAP amplicons and an automated data analysis (MSAP-Seq technique), more than 174 000 and 152 000 sites were analysed in the leaves and roots of the barley cv. ‘Karat’, respectively. We observed that, under control conditions, more than 9000 sites differed in the methylation level between leaves and roots. Most of these sites were methylated at a lower level in roots (80%) in comparison with leaves. GO enrichment analyses revealed that, within genes containing sites methylated at a lower level in roots, there was an over-representation of transmembrane transport, especially sulphate and nitrogen compound transport, post-embryonic organ development, and lipid and phosphorus metabolic processes, as well as the response to oxidative stress (see Supplementary Table S4 at *JXB* online). On the other hand, higher level of methylation in roots than in leaves was observed within DMSs in genes involved in many levels of the photosynthetic processes, regulation of some metabolic processes such as proline biosynthesis, and regulation of gene expression and other genes also engaged in transmembrane transport.

**Fig. 1.**
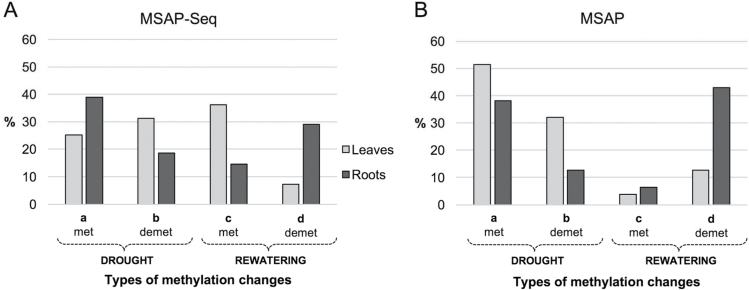
Percentage of the different types of DMSs that were identified using MSAP-Seq (A) and MSAP (B). (a) New methylation events under water deficiency; (b) demethylated under water deficiency; (c) unchanged under water deficiency, but methylated after recovery; (d) unchanged under water deficiency, but demethylated after recovery.

MSAP-Seq revealed 5000 different sites in leaves and 10 000 in roots that exhibited changes under either the water-deficiency treatment or subsequent rewatering. A random set of identified DMSs was initially validated with single-locus DNA methylation analysis using MSRE-qPCR and proved the reliability of detection of DMSs discovered by MSAP-Seq (data not shown). MSAP-Seq-based analysis of the DNA modulation types that occurred under water-deficiency stress and subsequent rewatering revealed a differential response of leaves and roots. Under water-deficiency treatment, more demethylation events were observed in leaves (30%) than in roots (8%), whereas novel methylations dominated in roots (40%) compared with leaves (25%) (Fig. 1A). Furthermore, we found many DMSs that underwent changes only during the rewatering phase; however, the novel methylations were more common in leaves (36% compared with 14% in roots), whereas and in roots demethylations dominated (7% in leaves and 29% in roots). A comparison of the observed types of changes in MSAP-Seq (Fig. 1A) with gel-based MSAP analysis (Fig. 1B) revealed many similarities as well as some differences between the results of these two technical approaches. The frequencies of identified demethylation events were comparable in MSAP and MSAP-Seq in both leaves and roots. Regarding new methylations, the results were similar in roots, but in leaves they were half as frequent in MSAP-Seq as in MSAP. The results of demethylation occurring under rewatering were again comparable between these two methods, but new methylations were much less frequently identified in MSAP than in MSAP-Seq. We presume that some of the observed discrepancies may result from the use of only one restriction enzyme (*Hpa*II) in MSAP-Seq instead of two (*Hpa*II and *Msp*I) in MSAP, as well as from the application of partially different PCR primers that might target various sites. Therefore, we have provided here the MSAP-based data mainly for the global estimation of DNA methylation in leaves and roots. The main conclusions of the comparative analyses of methylome changes upon water stress in leaves and roots were driven from data generated with the MSAP-Seq method.

### In contrast to demethylations, novel methylation events are mostly irreversible

**Fig. 2.**
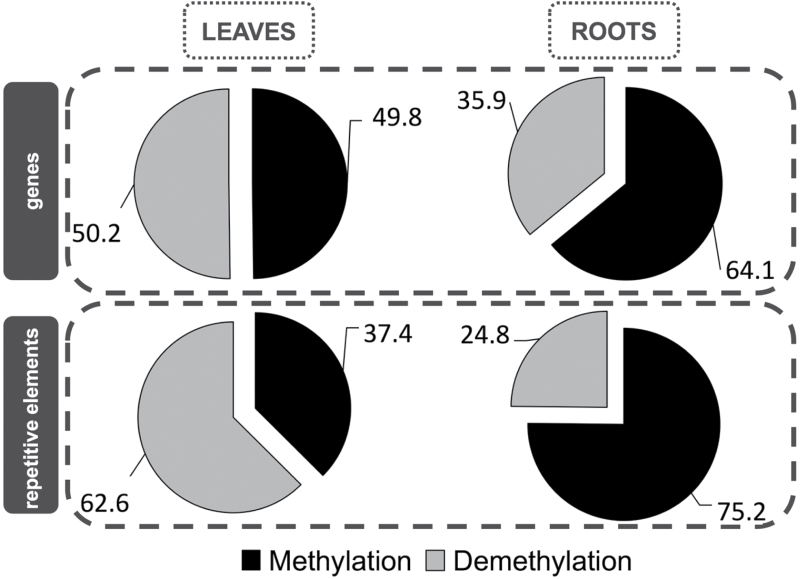
Percentages of the different types of DNA methylation changes that occurred in leaves and roots under water-deficiency stress within the genes and repetitive elements.

More detailed comparisons revealed interesting dissimilarities in leaves and roots regarding the type of DNA methylation changes that occurred within different genomic regions after water-deficit stress. Among the sites that were subjected to methylation changes, 950 in leaves and 2000 in roots were located within genes or their promoter regions, whereas 320 and 1500 in leaves and roots, respectively, were related to repetitive elements. First, we found that, in leaves, genes did not undergo any type of changes preferentially, and demethylations and novel methylations occurred with the same frequency (50:50). However, in roots, the new methylations that occurred in the genic regions were more prevalent than demethylations (65 vs 36%, respectively). In leaves, demethylations were more common (63%) than the methylation events (37%) among the DMSs that lay within the repetitive elements. Conversely, demethylations accounted for only one-quarter and novel methylation events for three-quarters of all DMSs in roots (Fig. 2).

**Fig. 3.**
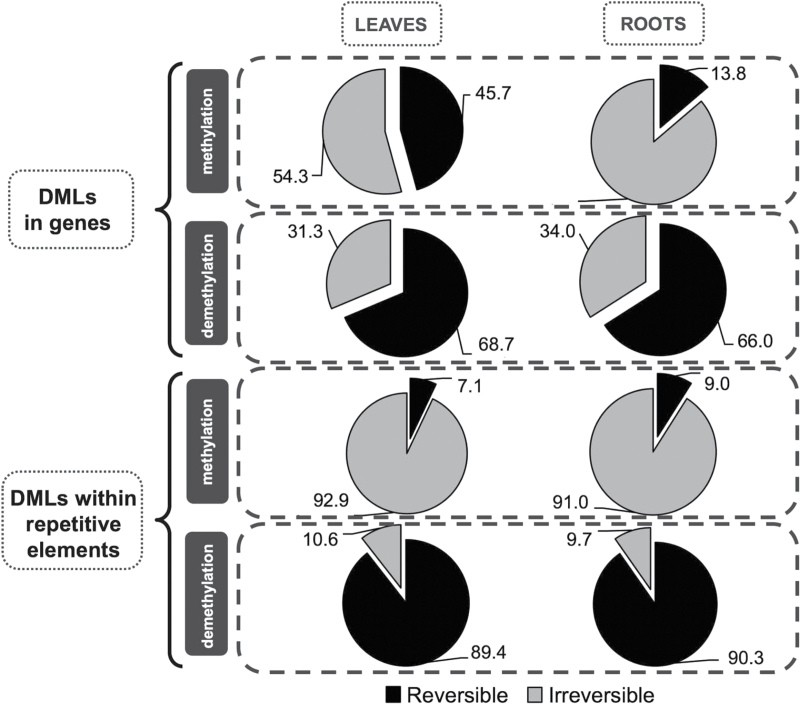
Percentages of reversible (during the rewatering phase) and irreversible types of DNA methylation changes that occurred under water deficiency in leaves and roots within the genes and repetitive elements.

Taking into account the differences among genic and repetitive element-related types of DMSs, we evaluated the reversibility pattern of novel methylations and demethylations in roots and leaves after a 2-week period of rewatering. We noticed that half of the new methylations that were located within genes were reversible in leaves, and that the other half was maintained during the rewatering phase. On the other hand, the majority of novel methylations were irreversible in roots (86%). However, in both of the analysed organs, as much as two-thirds of the gene-related demethylations were reversible and only one-third was irreversible. A quite distinct pattern was observed for the DMSs that were located in repetitive elements. A uniform pattern of reversible and irreversible changes was observed in both leaves and roots. The vast majority (>90%) of novel methylation events that were caused by water stress in repetitive sequences were irreversible upon rewatering, whereas demethylations were mostly reversible (Fig. 3).

**Fig. 4.**
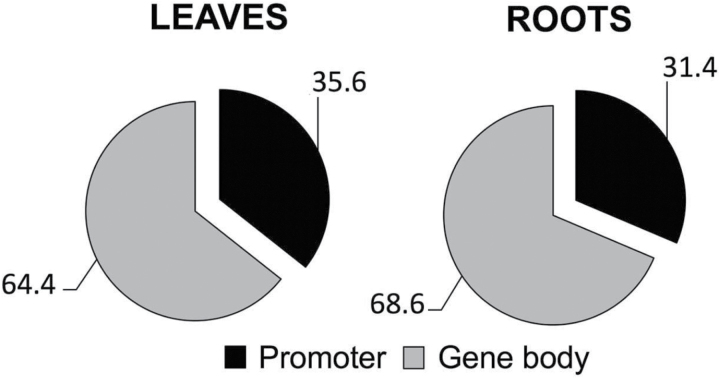
Percentages of gene-related features that underwent DNA methylation changes. Analyses correspond to DMSs that exhibited novel methylations or demethylations under water-deficiency treatment in roots and leaves.

Remarkably, we found that a much higher fraction of DNA methylation changes within genes was situated within the gene bodies than in the promoters. In both leaves and roots, around 65% of genic DMSs were related to the gene body and only 35% lay within the promoter regions (Fig. 4).

### Stress-related modulation of DNA methylation within the repetitive elements is similar in leaves and roots

Considering the lack of differences in the methylation pattern of repetitive elements in leaves and roots, we checked whether the diverse repetitive element groups were differentially targeted by the DNA methylation pathways in these organs. Due to the fact that annotation for repetitive elements in barley is far from being complete, and detailed information about transposons described by The International Barley Genome Sequencing Consortium (2012) are not publicly available, we could annotate only those transposons that are present in the Ensembl Plants database. Based on such partial, biased annotation, we could not precisely correlate any methylation data with the specific transposon frequency in the barley genome. The analysis of DMSs in leaves and roots revealed no significant differences in the participation of changes in the various repetitive element groups, regardless of the type of change. The most abundant of the repetitive elements with methylation changes were the long terminal repeat (LTR) retrotransposons, which contributed to almost 85–90% of DMSs in the repetitive DNA regions. The second group was the type II transposons, which formed around 10% of DMSs, while the rest accounted for less than 2–3% (see Supplementary Fig. S1 at *JXB* online). Taking into account the LTRs, we observed that similar LTR types underwent methylation changes in both organs with some slight differences in their frequencies. The most abundant type was Gypsy retrotransposons, which accounted for more than 20% in both organs. The second most frequent type was BARE in roots, also around 20%; however, BARE comprised only 9% of repetitive DMSs in leaves. SUKKULA and BAGY accounted for more than 10% in leaves and roots. The rest of the identified types constituted a similar percentage of participation in both organs (see Supplementary Table S3 at *JXB* online).

### Biological processes that are enriched in genic DMS datasets are disparate between roots and leaves

The identified genic DMSs, which were localized in the gene bodies or promoter regions, were analysed for GO enrichment. Determining which GO terms were significantly enriched within a genic DMS set can indicate the pathways that are most severely affected by DNA methylation under stress conditions. All of the specific GO terms enriched in the analysed subsets of genic DMS are presented in Supplementary Table S4. We focused on the processes that are specifically modulated by DNA methylation under water deficiency and hence might be important for stress response. Therefore, we analysed the sets of DMSs that underwent reversible alteration first. We found that among the genic DMSs that were reversibly methylated under water deficiency in leaves, there was an enrichment for processes such as xylogalacturonan metabolism, auxin influx, and several different developmental processes, whereas in the set of DMSs reversibly methylated under water deficiency in roots, there was an enrichment for processes such as anthocyanin accumulation in tissues in response to UV light, the positive regulation of calcium ion transport, protein import into the chloroplast thylakoid membrane, and some developmental and morphogenetic processes. Regarding the genic DMSs that were reversibly demethylated in leaves, there was a high over-representation of a wide range of biosynthetic processes, such as raffinose and the sphingolipid metabolic processes, as well as those connected with the response to cadmium ions and hexokinase-dependent signalling. In roots, this GO enrichment set included processes involved in the hypersensitive response, carbohydrate metabolism, and the positive regulation of developmental processes.

Next, we tested which biological processes were induced under water-deficiency conditions and persisted during the rewatering phase. Among the genes that underwent irreversible new methylations in leaves, we observed genes that were related to post-transcriptional gene silencing, auxin influx, and several metabolic and developmental processes. Interestingly, the processes connected to the response to stress were depleted. In roots, a different set of GO terms linked to the metabolic processes was found to undergo irreversible new methylations. It is noteworthy that a high level of enrichment for drought-recovery processes was observed in the irreversibly demethylated gene set in roots. In addition, we observed targeting for UV protection, a response to nutrient level and basic amino acid transport, as well as ATP biosynthesis.

Owing to the fact that our methylome analysis revealed that DNA methylation at some sites could be altered during the rewatering phase without any changes under water-deficiency, we assessed which processes were targeted by the epigenetic machinery during this phase. Among a methylated DMS set in leaves, we identified an enrichment for pectin catabolic processes, as well as calcium ion and auxin transport. When it came to the methylated genic DMS set in roots, it covered processes such as the response to mycotoxin, reactive oxygen and nitrogen species, and jasmonic acid metabolism. Interestingly, among the demethylated DMS set in roots, we found an enrichment for processes such as water transport, the sucrose biosynthetic process, the cellular response to oxidative stress, tissue development, and cytokinesis.

### Stress-related genes may undergo negatively related DNA methylation and expression changes in response to water-deficiency treatment

Because we observed a broad alteration of DNA methylation within the genic regions under water deficiency, we attempted to identify the water-deficiency-responsive genes at both the transcriptome and methylome levels that might suggest a potential contribution of epigenetic changes to gene expression modulation. We applied RT-qPCR to examine any differences in the expression level under control and water-deficiency conditions. A set of 36 genes that underwent DNA methylation changes under drought was randomly selected. The performed analysis revealed that 18 genes underwent negatively related DNA methylation and expression changes (Table 2). Two of the identified genes (MLOC_34020 and MLOC_62156) underwent demethylation within their gene body and transcriptional up-regulation under water deficiency in leaves. These genes may be potentially involved in the water-deficiency response as they encode stress-induced phosphoprotein 1 (MLOC_34020) and sucrose-phosphate synthase 2 (MLOC_62156). The rest of the 16 genes were subjected to novel methylation events within the gene body or promoter regions and the down-regulation of gene expression under water deficiency in leaves and/or roots. The identified genes were involved mainly in processes such as stress response, metabolism and oxidation–reduction reactions, protein modification, transport, cell division, photosynthesis, signal transduction, and transcription. For example, the gene that encodes the early-responsive to dehydration stress protein (MLOC_4742) underwent new methylations and the down-regulation of gene expression in roots. Moreover, we found two genes—MLOC_66056, which encodes the drug/metabolite transporter and MLOC_59937, which encodes the leucine-rich repeat family protein—which were both subjected to negatively coupled DNA methylation and gene-expression down-regulation in both roots and leaves.

**Table 2.** Comparison of relative methylation level determined with MSAP-Seq and gene expression modulation (RT-qPCR) under water deficiency in a subset of selected genes

**No.**	**Gene ID**	**Sample**	**Methylation changes upon drought (FC**)	**Genic localisation**	**Gene expression upon drought FC** ^***a***^	**Molecular function**	**Biological process**
1	MLOC_66056^*b*^	Leaves	M (24.0), R	Gene-body	6.3 down, R	Drug/metabolite Transporter	Transport
		Roots	M (12.2), IR	Gene-body	120.2 down, R		
2	MLOC_53364^*c*^	Roots	M (19.3), IR	Gene-body	2.1 down, IR	BEL1-like homeodomain protein	Transcription
			M (7.7), IR	Gene-body			
3	MLOC_59937^***b***^	Leaves	M (16.3), IR	Gene-body	10.0 down, R	Leucine Rich Repeat family protein	Other
		Roots	M (13.5), R	Gene-body	18.7 down, R		
4	MLOC_15252	Roots	M (16.2), IR	Gene-body	52.0 down, R	Amorpha-4,11-diene 12-monooxygenase	Oxidation- reduction process
5	MLOC_25536	Leaves	M (12.8), R	Gene-body	47.8 down, R	Anthocyanidin 5,3-O-glucosyltransferase	Metabolic process
6	MLOC_61723^*c*^	Leaves	M (11.5), R	Gene-body	224.4 down, R	Vesicular inhibitory amino acid transporter	Transport
			M (10.0), R	Gene-body			
			M (7.7), R	Gene-body			
7	MLOC_58032	Leaves	M (10.0), R	Gene-body	40.5 down, R	CENP-E like kinetochore protein	Cell division
8	MLOC_70149	Leaves	M (8.9), R	Promoter	540.3 down, R	Thebaine 6-O-demethylase	Oxidation- reduction process
9	AJ464414	Leaves	M (8.4), R	Gene-body	27.4 down, R	Jasmonate-induced protein	Stress response
10	MLOC_4742	Roots	M (7.3), IR	Gene-body	2.5 down, IR	Early-responsive to dehydration stress protein	Stress response
11	MLOC_36550	Leaves	M (6.4), R	Gene-body	5.2 down, IR	Small GTPase mediated Signal transduction	Signal transduction
12	MLOC_11877	Leaves	M (5.9), R	Gene-body	12.3 down, R	Chelatase subunit ChlI	Photosynthesis
13	AK252251	Leaves	M (4.3), R	Promoter	38.9 down, R	Cysteine proteinase	Protein modification
14	MLOC_44743	Leaves	M (4.0), R	Gene-body	117.5 down, R	NBS-LRR disease resistance protein-like	Stress response
15	morex_ contig_43608	Leaves	M (3.9), R	Gene-body	9.1 down, R	Unknown	Other
16	MLOC_10527	Leaves	M (3.6), R	Gene-body	7.9 down, R	Serine/threonine-protein kinase	Protein modification
17	MLOC_62156	Leaves	D (-12.3), R	Gene-body	3.0 up, R	Sucrose-phosphate synthase 2	Metabolic process
18	MLOC_34020	Leaves	D (-15.3), R	Gene-body	3.7 up, R	Stress-induced- phosphoprotein 1	Stress response

M, methylation; D, demthylation; R, reversible; IR, irreversible. Coloured shading indicates increased/decreased methylation or gene expression

(red to green, respectively).

*a* All values were statistically significant as determined by Student’s *t*-test (*P*≤0.05) with respect to the control.

*b* Different DMSs observed within the same gene in both leaves and roots.

*c* Two or three DMSs observed within one gene.

**Fig. 5.**
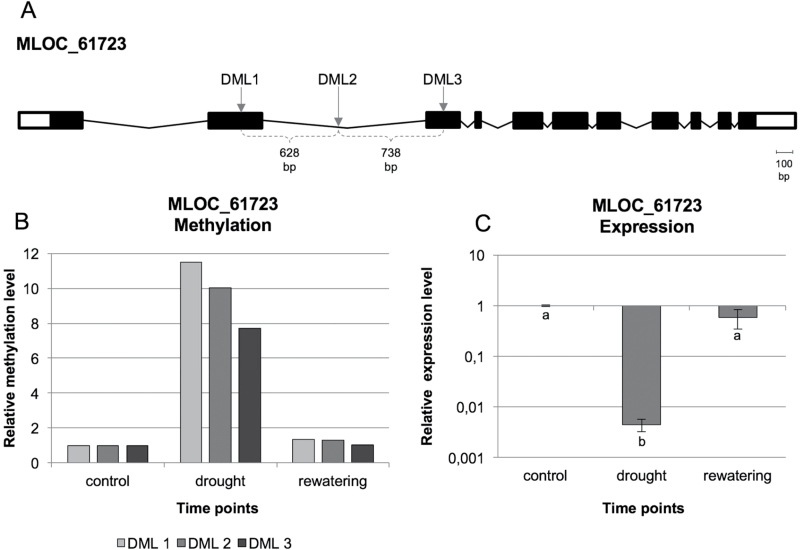
(A) Structure of the MLOC_61723 gene with the three DMSs that were identified indicated. (B) Relative methylation level of the three different DMSs in leaves, which were localized within the gene body of MLOC_61723 based on MSAP-Seq data. (C) Relative expression level of the MLOC_61723 gene in the leaves. Different letters indicate significant differences as determined by one-way ANOVA followed by Fisher’s LSD post-hoc test (*P*≤0.05). All values are presented as fold change (FC) in relation to the control (with FC=1).

We also identified genes that contained several DMSs, for example, MLOC_61723, which encodes the vesicular inhibitory amino acid transporter, and contains three DMSs within its gene body (Fig. 5). These DMSs covered around 1.4kb distance and were located evenly about 600–700bp from each other. All of them underwent new methylations under water deficiency and were demethylated back to their initial level after rewatering. The expression of MLOC_61723 decreased more than 200 times under water deficiency and returned to the basal level after the rewatering phase. A similar reversible pattern was observed for most of the 18 genes that were analysed, except for MLOC_36550 and MLOC_59937 in leaves, as well as MLOC_15252 and MLOC_66056 in roots. Moreover two genes in roots (MLOC_53364 and MLOC_4742) exhibited irreversible changes in DNA methylation and gene expression.

### Key genes that are involved in DNA methylation/demethylation pathways are transcriptionally modulated in leaves and roots

**Fig. 6.**
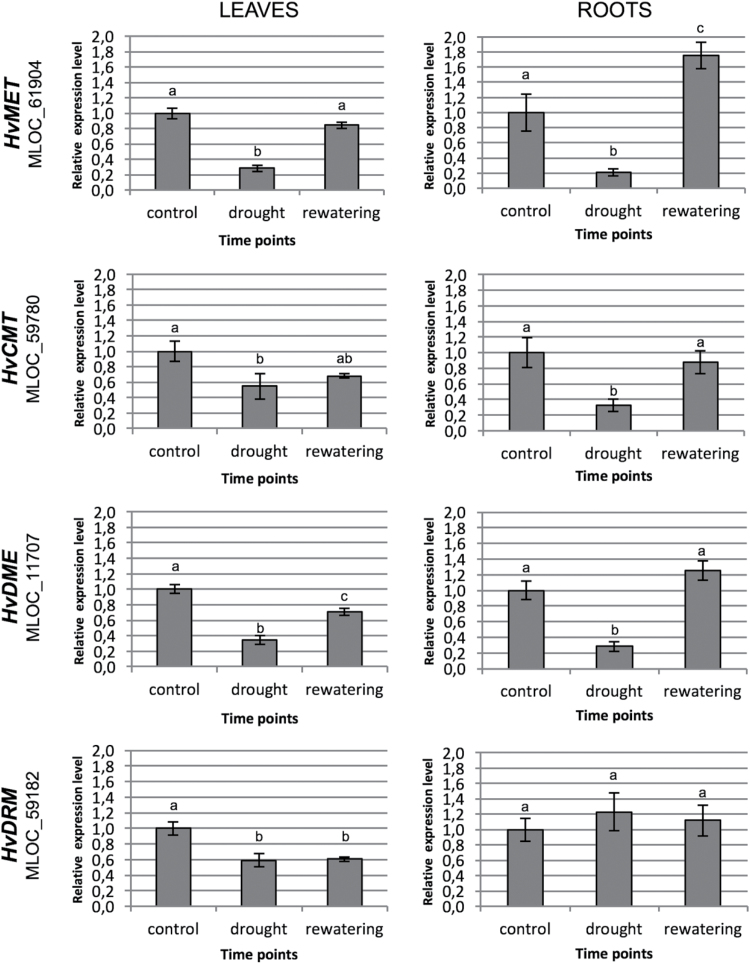
Relative expression level of *HvMET* (MLOC_61904), *HvCMT* (MLOC_59780), *HvDME* (MLOC_11707), and *HvDRM* (MLOC_59182) genes in leaves and roots. Different letters indicate significant differences as determined by one-way ANOVA followed by Fisher’s LSD post-hoc test (*P*≤0.05). All values are presented as fold change (FC) in relation to the control (with FC=1).

We evaluated the gene-expression level of four genes that are involved in the DNA methylation or demethylation machineries in barley: *HvMET* (*Hordeum vulgare METHYLTRANSFERASE*; MLOC_61904), *HvCMT* (*Hordeum vulgare CHROMOMETHYLASE*; MLOC_59780); *HvDRM* (*Hordeum vulgare DOMAINS REARRANGED METHYLTRANSFERASE*; MLOC_59182), and *HvDME* (*Hordeum vulgare DEMETER*; MLOC_11707). Transcriptional analysis using RT-qPCR revealed that of the three genes, *HvMET*, *HvCMT*, and *HvDME*, were down-regulated under water deficiency and returned almost to their initial level after rewatering in both leaves and roots. However, the *HvDRM* gene was observed to exhibit a different expressional pattern in these two organs. It was significantly down-regulated, almost 2-fold (*P*=0.00051), in leaves and did not return to its initial level after rewatering, and therefore the changed expression pattern persisted in the plants after recovery. On the other hand, *HvDRM* expression in roots was stable and did not change under water deficiency or during the rewatering phase (Fig. 6).

## Discussion

To the best of our knowledge, this study is the first comprehensive analysis of methylome modulation of barley leaves and roots in response to water-deficiency conditions and the subsequent rewatering phase. A global characterization of the barley methylome was carried out using the MSAP technique, which allows identification of the DNA methylation changes only within CCGG sequences; thus, the methylation states of the coupled CG and CHG context can be determined only within CCGG but not at the whole-genome level. The study revealed that overall level of DNA methylation within the analysed CCGG sequences was about 70%. This indicates a relatively high level of DNA methylation in comparison with other plants whose methylation level was estimated using the same method: 25% in *Arabidopsis thaliana* (Du and Wang, 2011), 20–30% in *Oryza sativa* (Karan *et al.*, 2012), 35% in *Zea mays* (Shan *et al.*, 2013), 50% in *Brassica napus* (Marconi *et al.*, 2013), and 70% in *Triticum aestivum* (Pan *et al.*, 2012). These comparisons suggest that the DNA methylation level corresponds with the genome size in plants, as a huge fraction of large genomes consists of repetitive sequences (mainly transposons); for example, in barley 84% of the genome is composed of them (International Barley Genome Sequencing Consortium, 2012). The recent evaluation of the methylcytosine content using high-performance liquid chromatography in phylogenetically independent representatives of angiosperms with different genome sizes has provided evidence for a positive correlation between the global DNA methylation level and the genome size (Alonso *et al.* 2015). Additionally, shotgun bisulphite sequencing revealed that the percentage of methylcytosines over total cytosines in a specific sequence context was 22% for CG and 6% for CHG in *A. thaliana*, 59% for CG and 21% for CHG in rice, and 86% for CG and 74% for CHG in maize (Feng *et al.* 2010; Gent *et al.*, 2013). The level of DNA methylation revealed in the presented results derives from the binomial state of the CCGG sequence, which represents cytosines in CG and CHG sequence contexts. Therefore, we suppose that in barley generally more than 70% of cytosines in the CG context might be present in the methylated state, and proportionally fewer cytosines should be methylated within CHG sequences.

The detailed analysis of DNA methylation changes that occurred under water deficiency and during the subsequent rewatering presented in this study allowed the precise identification of a wide range of DMSs in leaves and roots, which were located in non-coding as well as in gene-containing regions. However, the identified DMSs varied greatly between leaves and roots. We presumed that gene-related DMSs might play a potential role in the water-deficiency responses in both organs. Thus, by applying an analysis of GO enrichment, we attempted to determine what types of biological processes are the targets of the epigenetic machinery under water-deficiency stress and the subsequent rewatering phase. We observed an over-representation of various photosynthetic processes controlled by genes containing, under control conditions, highly methylated cytosines in roots but not in leaves. This observation suggests that the photosynthetic machinery may potentially be regulated at the DNA methylation level. We also identified many specific biological processes that were enriched in leaf- and root-specific DMS sets, which may result from organ-specific functions in response to water-deficiency stress. In the subset of reversible DMSs in leaves, we observed an enrichment of metabolic processes, such as xylogalacturonan and sphingolipid metabolism and raffinose biosynthesis, which may be involved in the mechanisms of the response and tolerance to the water-deficiency stress. Xylogalacturonan plays a role in cell-wall strengthening under abiotic stress conditions (Le Gall *et al.*, 2015), sphingolipids are involved in the signal-transduction pathway that leads to the reduction of turgor in guard cells, which in turn results in stomatal closure (Ng *et al.*, 2001), and raffinose acts as an osmoprotectant and confers a tolerance to water deficiency (Liu *et al.*, 2007). Moreover, an over-representation of processes that are linked to hexokinase-dependent signalling, which stimulates the stomatal closure and decreases transpiration, was also observed in the present study. This signalling pathway is also involved in the down-regulation of photosynthesis-related genes, which results in an inhibition of photosynthesis (Dai *et al.*, 1999; Rosa *et al.*, 2009; Kelly *et al.*, 2013). In roots, other processes that may be involved in the stress response were observed. For example, this group involved anthocyanin accumulation in tissues in response to UV light, protein import into chloroplast thylakoid membranes, and the regulation of calcium ion transport. Calcium gradients play a role in a sensory network by applying ion fluxes in the rapid transmission of information between distant sites in a plant (Choi *et al.*, 2014). Thus, calcium transport could be involved in the root-to-shoot signal transduction that leads to the induction of an adaptive response under water deficiency (Kiegle *et al.*, 2000).

Moreover, we observed that novel DNA methylation/demethylation events occurred upon rewatering. In leaves, for example, enrichment for pectin catabolic processes, basipetal auxin, and calcium and nickel ion transport were found, whereas in roots processes related to the response to mycotoxin, reactive oxygen and nitrogen species, jasmonic acid metabolism, and water transport were observed. This could suggest that plants probably deal with some consequences of water deficiency and adjust their metabolism and development to the new conditions after rewatering. The functional classification of genic DMSs and the performed analysis of biological process enrichment suggested the involvement of various developmental and metabolic-related pathways in leaves and roots under water-deficiency treatment and the following recovery. It can be supposed that the specific biological pathways that are affected by DNA methylation are disparate in leaves and roots; however, they are the result of the distinct but specialized functions of leaves and roots and together contribute to the stress-response networks.

It is noteworthy that, in spite of the different DNA modulations within gene-related sites between roots and leaves, we could draw the general conclusion that the water-deficiency conditions induced DNA methylation changes preferentially within the gene bodies rather than the promoter regions. Such a tendency was observed for both organs. The function of gene-body methylation has not yet been fully elucidated and different hypotheses have been established. It has already been reported that gene-body methylation has an even more repressive effect on transcription than the methylation within promoters (Vining *et al.*, 2012). Maunakea *et al.* (2010, 2013) suggested that body methylation might be involved in the suppression of expression from cryptic promoters that are localized within coding regions or modulation of alternative splicing. We identified a set of genes that underwent negatively coupled DNA methylation within their gene bodies and expression modulation in response to the water-deficiency treatment. This suggests that the transcriptional activity of at least some subsets of genes might be regulated through the alteration of gene-body methylation in response to abiotic stress. Although our study provides several examples of genes undergoing negatively coupled expressional and DNA methylation regulation under water-deficiency stress, it does not attempt to address any causality between abiotic stress, DNA methylation modulation, and gene expression changes on the global level.

The results of the detailed characterization of the barley methylome under water deficiency using the large-scale sequencing of MSAP amplicons allowed us to draw some general conclusions regarding the nature of changes that differentiate leaves and roots. The water-deficiency-related changes mostly involved demethylation events in leaves and new methylations in roots. This could be at least partially explained by the differential expression of the *HvDRM* gene, which is involved in RNA-dependent DNA methylation, in roots and leaves under water deficiency. In leaves, *HvDRM* was down-regulated, whereas in roots the transcriptional activity of *HvDRM* was not changed, and therefore the *de novo* methylation pathways were not inhibited. In addition to this, after rewatering, many novel methylations were induced in leaves, whereas there were de-methylations in roots. This indicated the presence of diverse and organ-specific responses to water limitation in the barley epigenome.

Interestingly, our results suggest some general differences regarding the type of DNA methylation modulation in different genomic regions. In roots, the novel methylations induced by water deficiency were more prevalent than demethylations, within both genic and repetitive elements-related regions. On the other hand, in leaves, the repetitive elements were mainly demethylated, whereas there was a similar proportion of methylation and demethylation events within the genes. The analysis of the fate of water-deficiency-induced cytosine methylation changes, both in the roots and the leaves, revealed that most of the demethylations were reversible upon rewatering, whereas the novel methylations were irreversible. Similar results were obtained in rice where demethylated sites returned to the previous status upon recovery (Wang *et al.*, 2011). In addition, we found that the changes that occurred within the repetitive elements were of a stricter nature than those in the genes with more than 90% of irreversible novel methylations or reversible demethylations. We suppose that such a tendency allows for a reversal of most of the demethylations that could have led to transposon mobilization and the maintenance of novel methylations that might silence them. We found that DMSs were located mainly within the LTR transposons, which are the most abundant in the barley genome, and that the distribution of the affected LTR types was similar in leaves and roots. This suggests that the water-deficiency-related methylation/demethylation machinery is not directed towards any of the transposon types that could be mobilized preferentially. On the other hand, the genic regions appeared to be subjected to the methylome modulation rather than an irreversible modification. This could suggest a role of the changes in the methylome that are induced by water deficiency in the direct regulation of the expression of water-deficiency-responsive genes.

Despite the existence of an evident methylome alternation, the overall DNA methylation level was stable and, in addition, was similar in leaves and roots, regardless of the growth conditions. The basis for this phenomenon has not yet been elucidated. Such DNA methylation stability could indicate that some kind of universal methylation-buffering mechanisms may be present, which might balance the overall methylation level to a certain range, even under severe stress conditions. Two recent studies in *A. thaliana* suggested that the *REPRESSOR OF SILENCING1* (*ROS1*) gene, which encodes an active DNA demethylase, could play an important role in the counteracting mechanisms (Lei *et al.*, 2015; Williams *et al.*, 2015). The authors suggested that *ROS1* functions as a ‘methylstat’ to sense DNA methylation levels, thereby tuning the demethylase activity through the regulation of its own expression. Such a mechanism would allow the dynamics of methylation and demethylation processes to be adjusted in response to methylation alterations in order to maintain the epigenomic stability.

In conclusion, this is the first study whose aim was to characterize barley methylome modulation in leaves and roots under water-deficiency stress and the subsequent rewatering phase. We established that the barley genome was highly methylated at a similar level in both leaves and roots; however, more hemi-methylations were observed in leaves, while full methylations were more abundant in roots. We observed that some of the methylation-related phenomena were similar in leaves and roots, whereas others exhibited striking differences between these two organs. The following assumptions regarding the differential responses of leaf and root methylomes to water-deficiency conditions are noteworthy:

1) In leaves, more demethylation than methylation events are induced under water deficiency, whereas novel methylations are more abundant in roots. The reversibility pattern of methylation changes also differs between the organs, with more reversible events being present in leaves than in roots. On the other hand, after the rewatering phase, more new methylations were induced in leaves, whereas there were more demethylations in roots.
2) Repetitive elements preferentially undergo demethylations in leaves and novel methylations in roots. A similar proportion of novel methylation and demethylation events were observed within genes in leaves and there was a domination of new methylations in roots.
3) The *HvDRM* gene, which is involved in *de novo* DNA methylation, is down-regulated under water deficiency in leaves, although its expression is not altered in roots.
4) Different biological processes are enriched within the subsets of leaf- and root-related DMSs that together contribute to the stress-response networks.

We presume that the organ specificity of methylome changes in response to water deficiency might be an important regulatory system that leads to the multi-level mechanisms of tolerance to stress in barley.

## Supplementary data

Supplementary data are available at *JXB* online.

**Fig. S1.** The percentages of different repetitive element groups that underwent changes in roots or leaves under water-deficiency stress and subsequent rewatering.

**Table S1.** The sequences of the primers and adapters that were used for the: A - MSAP analysis; B – MSAP-Seq analyses; C - RT-qPCR analysis.

**Table S2.** MSAP bands patterns corresponding to possible DNA methylation states.

**Table S3.** The percentages of different LTR types that underwent changes in roots or leaves under water-deficiency stress and subsequent rewatering.

**Table S4.** Gene ontologies of biological processes significantly enriched within the various subsets.

Supplementary Data

## References

[CIT0001] Alonso C Pérez R Bazaga P Herrera CM 2015 Global DNA cytosine methylation as an evolving trait: phylogenetic signal and correlated evolution with genome size in angiosperms. Frontiers in Genetics 6, 4. 25688257 10.3389/fgene.2015.00004 PMC4310347

[CIT0002] Boyko A Blevins T Yao Y Golubov A Bilichak A Ilnytskyy Y Hollander J Meins F. Jr Kovalchuk I 2010 Transgenerational adaptation of Arabidopsis to stress requires DNA methylation and the function of Dicer –like proteins. PLoS One 5, e9514. 20209086 10.1371/journal.pone.0009514 PMC2831073

[CIT0003] Braszewska-Zalewska AJ Wolny EA Smialek L Hasterok R 2013 Tissue-specific epigenetic modifications in root apical meristem cells of *Hordeum vulgare* . PLoS One 8, e69204. 23935955 10.1371/journal.pone.0069204 PMC3729647

[CIT0004] Candaele J Demuynck K Mosoti D Beemster GT Inzé D Nelissen H 2014 Differential methylation during maize leaf growth targets developmentally regulated genes. Plant Physiology 164, 1350–1364. 24488968 10.1104/pp.113.233312 PMC3938625

[CIT0005] Chen M Lv S Meng Y 2010 Epigenetic performers in plants. Development Growth and Differentiation 52, 555–566. 10.1111/j.1440-169X.2010.01192.x 20646028

[CIT0006] Choi WG Toyota M Kim SH Hilleary R Gilroy S 2014 Salt stress-induced Ca^2+^ waves are associated with rapid, long-distance root-to-shoot signaling in plants. Proceedings of the National Academy of Sciences, USA 111, 6497–6502. 10.1073/pnas.1319955111 PMC4035928 24706854

[CIT0007] Colaneri AC Jones AM 2013 Genome-wide quantitative identification of DNA differentially methylated sites in Arabidopsis seedlings growing at different water potential. PLoS One 8, e59878. 23577076 10.1371/journal.pone.0059878 PMC3620116

[CIT0008] Dai N Schaffer A Petreikov M Shahak Y Giller Y Ratner K Levine A Granot D 1999 Overexpression of Arabidopsis hexokinase in tomato plants inhibits growth, reduces photosynthesis, and induces rapid senescence. The Plant Cell 11, 1253–1266. 10402427 10.1105/tpc.11.7.1253 PMC144264

[CIT0009] Ding H Gao J Qin C Ma H Huang H Song P Luo X Lin H Shen Y Pan G Zhang Z 2014 The dynamics of DNA methylation in maize roots under Pb stress. International Journal of Molecular Sciences 15, 23537–23554. 25526567 10.3390/ijms151223537 PMC4284779

[CIT0010] Du Y Wang Z 2011 Methylation-sensitive amplified polymorphism analysis of DNA methylation in Arabidopsis under mannitol treatment. Chinese Bulletin of Botany 46, 285–292.

[CIT0011] Feng S Cokus SJ Zhang X 2010 Conservation and divergence of methylation patterning in plants and animals. Proceedings of the National Academy of Sciences, USA 107, 8689–8694. 10.1073/pnas.1002720107 PMC2889301 20395551

[CIT0012] Ferreira LJ Azevedo V Maroco J Oliveira MM Santos AP 2015 Salt tolerant and sensitive rice varieties display differential methylome flexibility under salt stress. PLoS One 10, e0124060. 25932633 10.1371/journal.pone.0124060 PMC4416925

[CIT0013] Filek M Janiak A Szarejko I Grabczyńska J Machácková I Krekule J 2006 Does DNA methylation pattern mark generative development in winter rape? Zeitschrift für Naturforschung C 61, 387–396. 10.1515/znc-2006-5-615 16869498

[CIT0014] Gayacharan Joel J 2013 Epigenetic responses to drought stress in rice (*Oryza sativa* L.). Physiology and Molecular Biology of Plants 19, 379–387. 24431506 10.1007/s12298-013-0176-4 PMC3715639

[CIT0015] Gent JI Ellis NA Guo L Harkess AE Yao Y Zhang X Dawe RK 2013 CHH islands: de novo DNA methylation in near-gene chromatin regulation in maize. Genome Research 23, 628–637. 23269663 10.1101/gr.146985.112 PMC3613580

[CIT0016] Guzy-Wrobelska J Kaliciak A Szarejko I Machackova I Krekule J Barciszewska M 2013 Vernalization and photoperiod-related changes in the DNA methylation state in winter and spring rapeseed. Acta Physiologiae Plantarum 35, 817–827.

[CIT0017] Harrison A Parle-McDermott A 2011 DNA methylation: a timeline of methods and applications. Frontiers in Genetics 2, 74. 22303369 10.3389/fgene.2011.00074 PMC3268627

[CIT0018] International Barley Genome Sequencing Consortium 2012 A physical, genetic and functional sequence assembly of the barley genome. Nature 491, 711–716. 23075845 10.1038/nature11543

[CIT0019] Karan R DeLeon T Biradar H Subudhi PK 2012 Salt stress induced variation in DNA methylation pattern and its influence on gene expression in contrasting rice genotypes. PLoS One 7, e40203. 22761959 10.1371/journal.pone.0040203 PMC3386172

[CIT0020] Kelly G Moshelion M David-Schwartz R Halperin O Wallach R Attia Z Belausov E Granot D 2013 Hexokinase mediates stomatal closure. Plant Journal 75, 977–988. 23738737 10.1111/tpj.12258

[CIT0021] Kiegle E Moore CA Haseloff J Tester MA Knight MR 2000 Cell-type-specific calcium responses to drought, salt and cold in the Arabidopsis root. The Plant Journal 23, 267–278. 10929120 10.1046/j.1365-313x.2000.00786.x

[CIT0022] Kou HP Li Y Song XX Ou XF Xing SC Ma J Von Wettstein D Liu B 2011 Heritable alteration in DNA methylation induced by nitrogen-deficiency stress accompanies enhanced tolerance by progenies to the stress in rice (*Oryza sativa* L.). Journal of Plant Physiology 168, 1685–1693. 21665325 10.1016/j.jplph.2011.03.017

[CIT0023] Le Gall H Philippe F Domon JM Gillet F Pelloux J Rayon C 2015 Cell wall metabolism in response to abiotic stress. Plants 4, 112–166. 10.3390/plants4010112 PMC4844334 27135320

[CIT0024] Lei M Zhang H Julian R Tang K Xie S Zhu J 2015 Regulatory link between DNA methylation and active demethylation in Arabidopsis. Proceedings of the National Academy of Sciences, USA 112, 3553–3557. 10.1073/pnas.1502279112 PMC4371987 25733903

[CIT0025] Lister R O’Malley RC Tonti-Filippini J Gregory BD Berry CC Millar AH Ecker JR 2008 Highly integrated single-base resolution maps of the epigenome in Arabidopsis. Cell 133, 523–536. 18423832 10.1016/j.cell.2008.03.029 PMC2723732

[CIT0026] Liu H Dai X Xu Y Chong K 2007 Over-expression of *OsUGE-1* altered raffinose level and tolerance to abiotic stress but not morphology in Arabidopsis. Journal of Plant Physiology 164, 1384–1390. 17566602 10.1016/j.jplph.2007.03.005

[CIT0027] Marconi G Pace R Traini A Raggi L Lutts S Chiusano M Guiducci M Falcinelli M Benincasa P Albertini E 2013 Use of MSAP markers to analyse the effects of salt stress on DNA methylation in rapeseed (*Brassica napus* var. oleifera). PLoS ONE 8, e75597. 24086583 10.1371/journal.pone.0075597 PMC3781078

[CIT0028] Maunakea AK Nagarajan RP Bilenky M 2010 Conserved role of intragenic DNA methylation in regulating alternative promoters. Nature 8, 253–257. 20613842 10.1038/nature09165 PMC3998662

[CIT0029] Maunakea AK Chepelev I Cui K Zhao K 2013 Intragenic DNA methylation modulates alternative splicing by recruiting MeCP2 to promote exon recognition. Cell Research 23, 1256–1269. 23938295 10.1038/cr.2013.110 PMC3817542

[CIT0030] Ng C Carr K McAinsh M Powell B Hetherington A 2001 Drought-induced guard cell signal transduction involves sphingosine-1-phosphate. Nature 410, 596–599. 11279499 10.1038/35069092

[CIT0031] Oakes CC La Salle S Robaire B Trasler JM 2006 Evaluation of a quantitative DNA methylation analysis technique using methylation-sensitive/dependent restriction enzymes and real-time PCR. Epigenetics 1, 146–152. 17965615 10.4161/epi.1.3.3392

[CIT0032] Ooi SK Bestor TH 2008 The colorful history of active DNA demethylation. Cell 133, 1145–1148. 18585349 10.1016/j.cell.2008.06.009

[CIT0033] Pan L Liu X Wang Z 2012 Comparative DNA methylation analysis of powdery mildew susceptible and resistant near–isogenic lines in common wheat. Life Science Journal 10, 2073–2083.

[CIT0034] Pfaffl MW 2001 A new mathematical model for relative quantification in real-time RT-PCR. Nucleic Acids Research 29, e45. 11328886 10.1093/nar/29.9.e45 PMC55695

[CIT0035] Proost S Van Bel M Vaneechoutte D Van de Peer Y Inzé D Mueller-Roeber B Vandepoele K 2015 PLAZA 3.0: an access point for plant comparative genomics. Nucleic Acids Research 43, D974–D981. 25324309 10.1093/nar/gku986 PMC4384038

[CIT0036] Ramakers C Ruijter JM Deprez RH Moorman AF 2003 Assumption-free analysis of quantitative real-time polymerase chain reaction (PCR) data. Neuroscience Letters 339, 62–66. 12618301 10.1016/s0304-3940(02)01423-4

[CIT0037] Rosa M Prado C Podazza G Interdonato R González J Hilal M Prado F 2009 Soluble sugars-metabolism, sensing and abiotic stress: a complex network in the life of plants. Plant Signaling & Behavior 4, 388–393. 19816104 10.4161/psb.4.5.8294 PMC2676748

[CIT0038] Shan X Wang X Yang G Wu Y Su S Li S Li H Yuan Y 2013 Analysis of the DNA methylation of maize (*Zea mays* L.) in response to cold stress based on methylation–sensitive amplified polymorphisms. Journal of Plant Biology 56, 32–38.

[CIT0039] Song Y Jo D Li S Wang P Li Q Xiang F 2012 The dynamic changes of DNA methylation and histone modifications of salt responsive transcription factor genes in soybean. PLoS One 7, e41274. 22815985 10.1371/journal.pone.0041274 PMC3399865

[CIT0040] Tang XM Tao X Wang Y Ma DW Li D Yang H Ma XR 2014 Analysis of DNA methylation of perennial ryegrass under drought using the methylation-sensitive amplification polymorphism (MSAP) technique.Molecular Genetics and Genomics 289, 1075–1084. 24916310 10.1007/s00438-014-0869-6

[CIT0041] Vining KJ Pomraning KR Wilhelm LJ Priest HD Pellegrini M Mockler TC Freitag M Strauss SH 2012 Dynamic DNA cytosine methylation in the Populus trichocarpa genome: tissue-level variation and relationship to gene expression.BMC Genomics 13, 27. 22251412 10.1186/1471-2164-13-27 PMC3298464

[CIT0042] Wang WS Pan YJ Zhao XQ Dwivedi D Zhu LH Ali J Fu BY Li ZK 2011 Drought-induced site-specific DNA methylation and its association with drought tolerance in rice (*Oryza sativa* L.). Journal of Experimental Botany 62, 1951–1960. 21193578 10.1093/jxb/erq391 PMC3060682

[CIT0043] Wang M Qin L Xie C Li W Yuan J Kong L Yu W Xia G Liu S 2014 Induced and constitutive DNA methylation in a salinity-tolerant wheat introgression line. Plant and Cell Physiology 55, 1354–1365. 24793752 10.1093/pcp/pcu059

[CIT0044] Williams B Pignatta D Henikoff S Gehring M 2015 Methylation-sensitive expression of a DNA demethylase gene serves as an epigenetic rheostat. PLoS Genetics 11, e1005142. 25826366 10.1371/journal.pgen.1005142 PMC4380477

[CIT0045] Xu M Li X Korban S 2000 AFLP-based detection of DNA methylation. Plant Molecular Biology Reporter 18, 361–368.

[CIT0046] Zhang M Xu C Wettstein D Liu B 2011 Tissue-specific differences in cytosine methylation and their association with differential gene expression in sorghum. Plant Physiology 156, 1955–1966. 21632971 10.1104/pp.111.176842 PMC3149958

