# Supplementary material for: Water-deficiency conditions differently modulate the methylome of roots and leaves in barley (Hordeum vulgare L.)
Source: J Exp Bot. 2016 Jan 5;67(4):1109–21. doi: 10.1093/jxb/erv552 (PMC4753852; doi:10.1093/jxb/erv552)
Supplement: Supplementary Data [file supp_67_4_1109__index.html]

Water-deficiency conditions differently modulate the methylome of roots and leaves in barley (Hordeum vulgare L.) — Water-deficiency conditions differently modulate the methylome of roots and leaves in barley (Hordeum vulgare L.) — Supplementary Data 

# Water-deficiency conditions differently modulate the methylome of roots and leaves in barley (*Hordeum vulgare* L.)

## Supplementary Data

Data files

- supplementary\_figure\_S1\_tables\_S2\_S3.pdf - Supplementary Data
- Supplementary\_table\_S1.xlsx - Supplementary Data
- Supplementary\_table\_S4.xlsx - Supplementary Data
