# Supplementary material for: Water-deficiency conditions differently modulate the methylome of roots and leaves in barley (Hordeum vulgare L.)
Source: J Exp Bot. 2016 Jan 5;67(4):1109–21. doi: 10.1093/jxb/erv552 (PMC4753852; doi:10.1093/jxb/erv552)
Supplement: Supplementary Data [file supp_erv552_supplementary_figure_S1_tables_S2_S3.pdf]

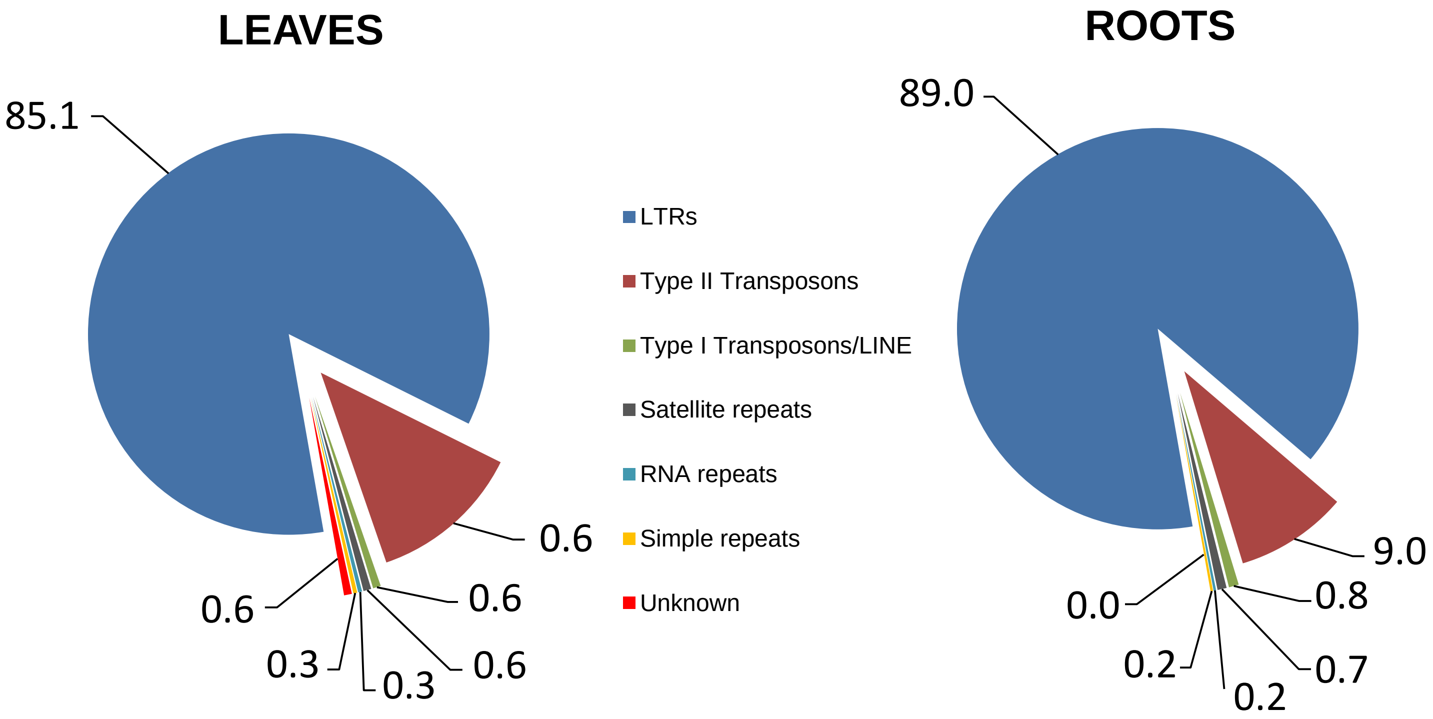

Supplementary Figure S1. The percentages of different repetitive element groups that underwent changes in roots or leaves under water deficiency stress and subsequent rewatering.

Supplementary Table S2. MSAP bands patterns corresponding to possible DNA methylation states

Band pattern	*Hpa*II	*Msp*I	Type	Methylation state
**I**	1	1	Demethylated or hemimethylated inner C	CCGG or CCGG<br>GGCC GGCC
**II**	1	0	Hemimethylated outer C or one strand hemimethylated	CCGG or CCGG<br>GGCC GGCC
**III**	0	1	Fully methylated inner C	CCGG<br>GGCC
**IV**	0	0	Fully methylated or fully methylated outer C or fully methylated outer C + hemimethylated inner C or hemimethylated outer C + fully methylated inner C	CCGG or CCGG or CCGG or CCGG<br>GGCC GGCC GGCC GGCC

Supplementary Table S3. The percentages of different LTR types that underwent changes in roots or leaves under water deficiency stress and subsequent rewatering

LEAVES		ROOTS	
**Group**	**%**	**Group**	**%**
Gypsy	29.4	Gypsy	22.8
SUKKULA	15.6	BARE	21.6
BAGY	11.9	SUKKULA	13.3
BARE	8.9	BAGY	11
Copia	8.9	Maximus	8.3
Maximus	7.4	SABRINA	7.3
SABRINA	6.7	Copia	5.3
INGA	4.1	INGA	3.8
CEREBA	2.6	CEREBA	3.6
IKEROS	1.9	NIKITA	0.9
INAV	1.1	OPHELIA	0.7
OPHELIA	0.7	INAV	0.6
Unknown	0.7	IKEROS	0.5
NIKITA	0	Unknown	0.3
